# 118. Machine Learning Approaches to Predicting Treatment Outcomes for Carbapenem-Resistant Enterobacterales in a Region with High Prevalence of Non-Carbapenemase Producers

**DOI:** 10.1093/ofid/ofab466.118

**Published:** 2021-12-04

**Authors:** Cody A Black, Samantha Aguilar, Sarah Bandy, Gerard Gawrys, Steven Dallas, Wonhee So, Raymond Benavides, Neha Betrabet, Catherine Diaz, Audrey Anderson, Grace Lee

**Affiliations:** 1 UT AUSTIN, San Antonio, TX; 2 University of Texas at Austin College of Pharmacy, San Antonio, TX; 3 Methodist Hospital & Methodist Children's Hospital, San Antonio, Texas; 4 UT Health San Antonio, SAN ANTONIO, Texas; 5 New York Presbyterian, New York, New York; 6 The University of Texas at Austin College of Pharmacy, Pharmacotherapy Education and Research Center, School of Medicine, UT Health San Antonio, San Antonio, Texas

## Abstract

**Background:**

Carbapenem-resistant Enterobacterales are a growing threat globally. Early detection of CRE is necessary for appropriate treatment and infection control measures. Many hospital labs can test for carbapenemase production; however, in some regions, including South Texas, the majority of CRE are non-carbapenemase producing (NCPE). This study had two interrelated aims to develop decision rules tailored to a region with high prevalence of NCPE to predict 1) antimicrobial resistance (AMR) from whole genome sequencing (WGS) data and 2) CRE treatment outcomes.

**Methods:**

To better understand links between resistome, phenotypic AMR, and prediction of outcomes for CRE, we developed decision rules to build machine learning prediction models. We conducted WGS and antibiotic susceptibility testing (21 antibiotics) on CRE isolates from unique patients across 5 hospitals in the South Texas region between 2013 and 2020. Day 30 outcomes were based on desirability of outcome ranking (DOOR). The overall classification accuracies of the models are reported.

**Results:**

Overall 146 CRE isolates were included, 97 were used to train each model, and 49 were used for validation. Among the *K. pneumoniae* and *E. coli* CRE isolates that were available with susceptibility data, the majority (62%) were NCPE. For the clinical recovery model (DOOR), the combination of admission ICU status, piperacillin-tazobactam (PT) MIC > 16, presence of *sul* gene, and polymyxin-sparring regimens associated with an overall accuracy of 95% for having a worse DOOR. Majority (60%) of patients were empirically treated with piperacillin-tazobactam; notably, less than 33% isolates had PT MIC ≤ 16. Interestingly, combined effects of isolates that did not harbor carbapenemases, blaOXA-1, blaCTX-M-15, blaCMY, or aac(6’)ib-cr genes resulted in a decision rule with a 95.7% overall accuracy for susceptibility to PT (MIC < 16 ug/mL).

**Conclusion:**

Herein, we used machine learning approaches to predict AMR and treatment-based outcomes linked with WGS data in a region with predominantly NCPE infections. Machine learning can obtain a model that can make repeatable predictions, further data can improve the accuracy and guide tailored clinical decision-making.

**Disclosures:**

**Grace Lee, PharmD, PhD, BCPS**, **Merck Co.** (Grant/Research Support)**NIA/NIH** (Research Grant or Support)

